# Neuropsychopharmacology renaissance in Japan: A new era after the crisis

**DOI:** 10.1111/pcn.13549

**Published:** 2023-04-13

**Authors:** Norio Ozaki, Teiji Kimura, Tetsuro Kikuchi, Takeo Ishiyama

**Affiliations:** ^1^ Pathophysiology of Mental Disorders, Nagoya University Graduate School of Medicine Nagoya Japan; ^2^ Eisai Co., Ltd. Tokyo Japan; ^3^ Otsuka Pharmaceutical Co., Ltd. Tokushima Japan; ^4^ Drug Research Division, Sumitomo Pharma Co., Ltd. Osaka Japan

In 2011, an Editorial in *Nature* cited statements from several societies, including the European College of Neuropsychopharmacology, sounding the alarm on psychopharmacology in crisis.^1^

Diagnosing and evaluating mental disorders are based on mental symptoms, not objective test data. Furthermore, the pathophysiology of mental disorders must be elucidated in the brain, a complex organ explicitly in human beings. As a result, the elucidation of the pathophysiology of mental disorders has been delayed, and it is difficult to stratify and identify drug targets based on the pathophysiology. Due to the above, developing therapeutic drugs for mental disorders requires much time and money, but the probability of success is low. In this situation, around 2010, several big pharmaceutical companies involved in central nervous system drug discovery underwent a major restructuring, cutting research funding in this area and closing teams dedicated to developing drugs for mental disorders.

After the crisis in psychopharmacology was reported a decade ago, how have the major pharmaceutical companies been working on novel drug development in this field? To get insight into the answer to the question, we have counted the number of novel drug candidates for mental disorders and neurological disorders under clinical phase 3 study or novel drug application by large pharmaceutical companies (total 79 companies with R&D expenses of US$500 million or more among the top 1000 in annual sales (source by GlobalData)), referring to their latest publication of drug pipelines from April 2022 to January 2023. The analysis included 17 Japan‐based companies (average R&D costs of US$1335M) and 62 other companies (US$2971M). These companies developed a total of 51 novel drug pipelines, seven of which were jointly developed, resulting in a total of 43 different drugs. Of these, eight were for indications for mental disorders, and 35 were for neurological disorders. Of the 51 drug pipelines, 19 and 32 were developed by eight and 14 pharmaceutical companies headquartered in Japan and others, respectively. These drugs under development include two FDA breakthrough therapy designations in psychiatric disorders and six in neurological disorders, such as ulotaront for schizophrenia^2^ and lecanemab for Alzheimer's disease.^3^ The total eight designations involved six Japan‐based companies and six others, including four co‐development.

The analysis found that co‐development among the analyzed companies is one of the ways to develop novel drugs targeting mental disorders and neurological disorders. Furthermore, the over‐representation of Japan‐based companies among all analyzed ones developing novel drugs and those with breakthrough therapy designations (*P* = 0.067 and 0.018, respectively, by Fisher's exact test, see Fig. 1) shows that Japan‐based ones are playing a major role in the development of novel drugs for mental disorders and neurological disorders. Although it cannot be ruled out that the difference may reflect that in strategies of the pharmaceutical companies between Japan‐based ones and others, for example a different way of collaborating with venture companies, these data may indicate that Japan‐based ones have continued their efforts to find clues for drug innovation even during the crisis in this field.

**Fig. 1:**
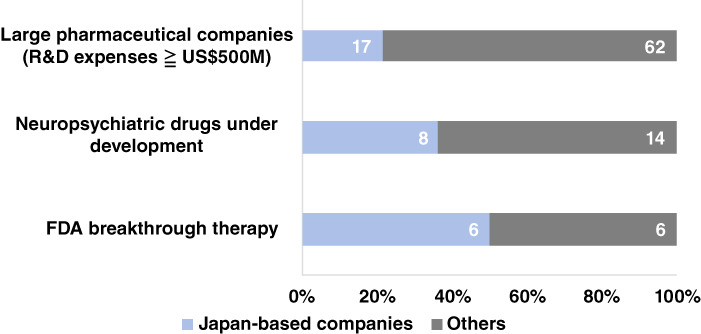
Rates of neuropsychiatric drugs developed by companies headquartered in Japan.

Given the huge socioeconomic burden, we hope more pharmaceutical companies will become involved in drug discovery, especially for mental disorders. To improve success probability, industry‐academia collaboration is indispensable for elucidating the pathophysiology of mental disorders and developing treatments based on the pathophysiology.

We hope the neuropsychopharmacology renaissance in Japan, a new era after the crisis, will answer the wish of patients with mental illness and their families.

## References

[1] Cressey D . Psychopharmacology in crisis. Nature 2011. 10.1038/news.2011.367.

[2] Koblan KS , Kent J , Hopkins SC *et al*. A non‐D2‐receptor‐binding drug for the treatment of schizophrenia. N. Engl. J. Med. 2020; 382: 1497–1506. 32294346 10.1056/NEJMoa1911772

[3] van Dyck CH , Swanson CJ , Aisen P *et al*. Lecanemab in early Alzheimer's disease. N. Engl. J. Med. 2023; 388: 9–21. 36449413 10.1056/NEJMoa2212948

